# Capsaicin combined with dietary fiber prevents high‐fat diet associated aberrant lipid metabolism by improving the structure of intestinal flora

**DOI:** 10.1002/fsn3.3043

**Published:** 2022-09-20

**Authors:** Ting Gong, Yujing Zhou, Lei Zhang, Haizhu Wang, Min Zhang, Xiong Liu

**Affiliations:** ^1^ College of Food Science Southwest University Chongqing People's Republic of China; ^2^ Chongqing Medical and Pharmaceutical College Chongqing People's Republic of China; ^3^ College of Life Science Chongqing Normal University Chongqing People's Republic of China

**Keywords:** capsaicin, dietary fibers, intestinal microbiome, lipid metabolism

## Abstract

Capsaicin (CAP) and dietary fibers are natural active ingredients that given separately do positively affect obesity and metabolic diseases. However, it was unknown whether their combined administration might further improve blood lipids and gut flora composition. To test this hypothesis we administered capsaicin plus dietary fibers (CAP + DFs) to male rats on a high‐fat diet and analyzed any changes in the intestinal microbiota make up, metabolites, and blood indexes. Our results showed that combining CAP with dietary fibers more intensely reduced total cholesterol (TC) and low‐density lipoprotein cholesterol (LDL‐C). CAP + DFs also increased gut bacteria variety, and the abundance of several beneficial bacterial strains, including *Allobaculum* and *Akkermansia*, while reducing harmful strains such as *Desulfovibrio.* Additionally, CAP + DFs significantly increased arginine levels and caused short‐chain fatty acids accumulation in the contents of the cecal portion of rats' gut. In conclusion, notwithstanding the rats were kept on a high‐fat diet, adding CAP + DFs to the chow further improved, as compared with CAP alone, the lipidemia and increased the gut beneficial bacterial strains, while reducing the harmful ones.

## Graphical abstract

In conclusion, notwithstanding the rats were fed a high‐fat diet, adding CAP plus fibers to the chow improved the lipidemia and increased the gut beneficial bacterial strains, while reducing the harmful ones as compared with CAP alone.

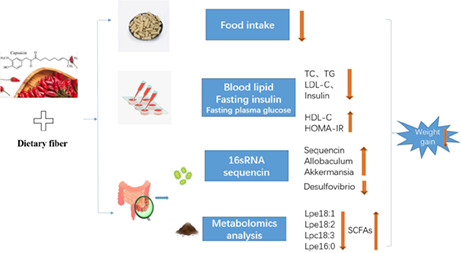

## 1 INTRODUCTION

During the 21st century, China has been experiencing an era of rapid nutritional transformation. The traditional Chinese diet, rich in carbohydrates and vegetable fibers, has been increasingly replaced by refined foods and a more Western‐style diet (Tian & Wang, 2019). These steadily spreading eating habits, entailing the consumption of refined saturated fat‐rich foods as contrasted with vegetable fibers are promoting obesity and metabolic syndromes/diseases (dyslipidemias, insulin resistance, diabetes), steatohepatitis, and cardiovascular illnesses (Hosoi et al., 2016; Lindroos et al., 2021).

Typically, microbes in the cecum and colon portions of the gut metabolize vegetable fibers into acetic, propionic, and butyric acids, altogether indicated as short‐chain fatty acids (SCFAs) (Barber et al., 2020; Cheng et al., 2020). In their turn, SCFAs interact with enteroendocrine L cell receptors whose activation induces the tyrosine–tyrosine (PYY) peptide release (Farzi et al., 2021).

Almost all chillies synthesize CAP, which is responsible of their characteristic pungent flavor. CAP significantly regulates intestinal microbiome and exerts beneficial anti‐inflammatory, lipid‐lowering, and hypoglycemic effects (Rosca et al., 2020; Zhang et al., 2019). However, CAP's efficacy is conditioned by its dose and time of action. In an earlier study, we showed that CAP could decrease food intake, weight gain, fasting plasma triglycerides (TG), total cholesterol (TC), glucose, and insulin (Gong et al., 2022; Wang, Tang, et al., 2020). Moreover, CAP improved insulin effectiveness and increased SCFAs and the abundance of bacterial strains, for example, the *Akkermansia* genus, mitigating high‐fat diet‐induced metabolic abnormalities (Hui et al., 2019; Rosca et al., 2020; Shen et al., 2017). Simultaneously, CAP increased the gut plentifulness of *Desulfovibrio* genus bacteria, which promotes colitis development (Chen et al., 2019). Therefore, we resolved to investigate whether combining CAP with other natural ingredients in the diet might: (1) improve glucose and lipid metabolism; and (2) decrease the abundance of pathogenic bacteria further than did CAP alone.

## 2 EXPERIMENTAL METHODS

### 2.1 Materials

CAP (≥95% purity) was from Henan Bis‐Biotech Co., Ltd. Inulin (≥90% purity) was from Henan Wanbang Industrial Co., Ltd.

### 2.2 Animals and treatments

All the standard operating procedures for the animal experiments strictly abided by the EU Directive 2010/63/EU. The Institutional Animal Care and Use Committee of Southwest University approved the protocols (IACUC‐20201008‐01). Male Sprague–Dawley (SD) rats (200 ± 20 g) (from Hunan SJA Laboratory Animal Co., Ltd) were kept inside individual stainless steel cages at 25 ± 1°C, with a 40%–70% a relative humidity and a 12 h light/dark cycle. In the week preceding the experiments, rats had an ad libitum access to water and were kept on a standard diet. A total of 30 rats were randomly divided into three groups (*n* = 10): (i) high‐fat diet (HF); (ii) CAP 0.01% + high‐fat diet (HFC); (iii) CAP0.01% + 5%DFs + high‐fat diet (HFBM). Table S1 lists each experimental group's diet ingredients. The high‐cholesterol food contained a 1% cholesterol supplement based on U.S. Standard AIN‐93 feed formula (Afrose et al., 2009). Food treated with Co^60^ irradiation for sterilization was purchased from the Jiangsu Xietong Pharmaceutical Bio‐engineering Co., Ltd. The sterilized formulations were stored in a refrigerator (−20°C) until use.

### 2.3 Sample collection

The rats' survival and food intake were checked every day. The rats’ weight was recorded once weekly. After 4 weeks of feeding, rats were fasted for one day and next sacrificed. Immediately after sacrifice, their blood, cecum with its contents, and small intestine with its contents were collected under aseptic conditions. All samples were immediately placed in sterile tubes, flash‐frozen (30 s) in liquid N, and next stored at −80°C.

### 2.4 Blood biochemical analyses

Plasma triglycerides (TG) and cholesterol (TC) levels were determined via commercial kits (BioSino, Beijing, China). Fasting plasma glucose (FPG), high‐density lipoprotein cholesterol (HDL‐C), and low‐density lipoprotein cholesterol (LDL‐C) were measured in an autoanalyzer (URIT‐8021A). Plasma fasting insulin was assessed via a Rat Insulin (INS) ELISA Kit (Sinobestio, Shanghai, China).

### 2.5 Gas chromatographic (GC) SCFAs' quantification

SCFAs' levels of the rats’ cecal contents were determined using an Agilent 7890 A according to the GC method previously described by Zhang (2013). Chromatographic conditions were as follows: injection volume, 1.0 μl; inlet temperature, 220°C; column flow, 0.95 ml/min; column temperature, 90°C; equilibrium time, 0.5 min; temperature increased to 150°C by 5°C/min; retention time, 9 min; detector temperature, 230°C; hydrogen flow, 40 ml/min; air flow, 400 ml/min; and tail blowing flow, 40 ml/min.

The retention time of each chromatographic peak was used for qualitative analysis, and the resulting peak areas for different standard concentrations served for the quantitations. The GC chromatogram of SCFA standards is shown in Figure S1, while the standard curves are provided in Table S2.

### 2.6 Gut microbiota analysis by 16S rRNA gene sequencing

DNA preparation, PCR amplification, and pyrosequencing were performed according to Kørner et al. (2015). Briefly, total DNA was extracted from 250 mg of cecal contents using E.Z.N.A. Stool DNA kit (OMEGA, Bio‐Tek, USA). To identify the gut microbiota a primer set (515F/926R) served to amplify the 16S rRNA gene V4 region. Each sample was amplified in Library preparation and pyrosequencing was performed on the 454 GS FLX sequencing platform (Roche) at the Chinese National Human Genome Center in Shanghai. The raw sequences are available via the NCBI/EBI/DDBJ Sequence Read Archive (Accession Nos. DRA002627 and DRA0012641).

The analysis of all cecal contents was conducted by means of the Illumina HiSeq platform (insert size, 250–450 bp; read length, 250 bp). The 30,681 sequences obtained from each sample could be separately rarefied, so that the sampling workload of the entire sample was equalized. To foretell the microbial communities function profiles, an operational taxonomic unit (OTU) analysis was conducted by clustering the sequences at a 97% similarity level (USEARCH, version 10.0). OTUs were filtered based on a threshold set at 0.005% for all sequences. The Shannon Index, Bray–Curtis Distance, and principal component analysis (PCA) results were plotted using R software (Version 3.4.0 by Vegan Package).

### 2.7 LC–MS/MS metabolites analysis

A 100‐mg sample of rat cecal contents was weighed and mixed thoroughly in an 800 μl acetonitrile/methanol (1:1 w/v) mixture, which was next incubated for 60 min at −20°C and thereafter centrifuged at 16,000 rpm at 4°C for 20 min. The upper phase was collected, vacuum‐dried, and next added to 100 μl acetonitrile/water (1:1 w/v) for analysis in a 1290 Infinity LC system (Agilent, USA) linked to a Pegasus HT TOF mass spectrometer (LECO, USA). An ACQUITY UPLC BEH Amide 1.7 μm, 2.1 mm × 100 mm column was served to separate the single compounds. A 5.0 μl injection volume and a 25°C column temperature were used. The flow rate was 0.3 ml/min.

LC‐TOF/MS raw data were first analyzed by MSDIAL software using the XCMS database, including raw peak extraction, data baseline filtering and calibration, peak alignment, deconvolution analysis, peak identification, and peak area integration. Subsequently, normalized data were input into the SIMCA 14.1 software package (Umetrics AB, Umea, Sweden). After centralizing means and scaling unit variances, multivariate analyses were performed, such as orthogonal partial least squares discriminant analysis (OPLS‐DA), and principal component analysis (PCA), to visualize metabolic differences between the three groups. Eventually, searching the Kyoto Encyclopedia of Genes and Genomes (KEGG, http://www.genome.jp/kegg/) allowed to characterize the differentially expressed metabolites.

### 2.8 Statistical analysis

The homeostatic model assessment–insulin resistance (HOMA‐IR) index was calculated via the formula: HOMA‐IR = fasting insulin × fasting glucose/22.5 (Huang et al., 2017; Lin et al., 2019; Wang, Lu, et al., 2020). Each treatment group had at least three replicates (*n* = 3), and each experiment was repeated three times. All data values were expressed as means ± standard deviations. One‐way analysis of variance was performed using SPSS version 20.0 and Origin 8.5 software. Duncan's multiple‐range test served to assess any differences among groups. Values of *p* < .05 were taken as statistically significant.

## 3 RESULTS

### 3.1 Effects of CAP + DFs on food intake and body weight of rats on high‐fat diet

**FIGURE 1:**
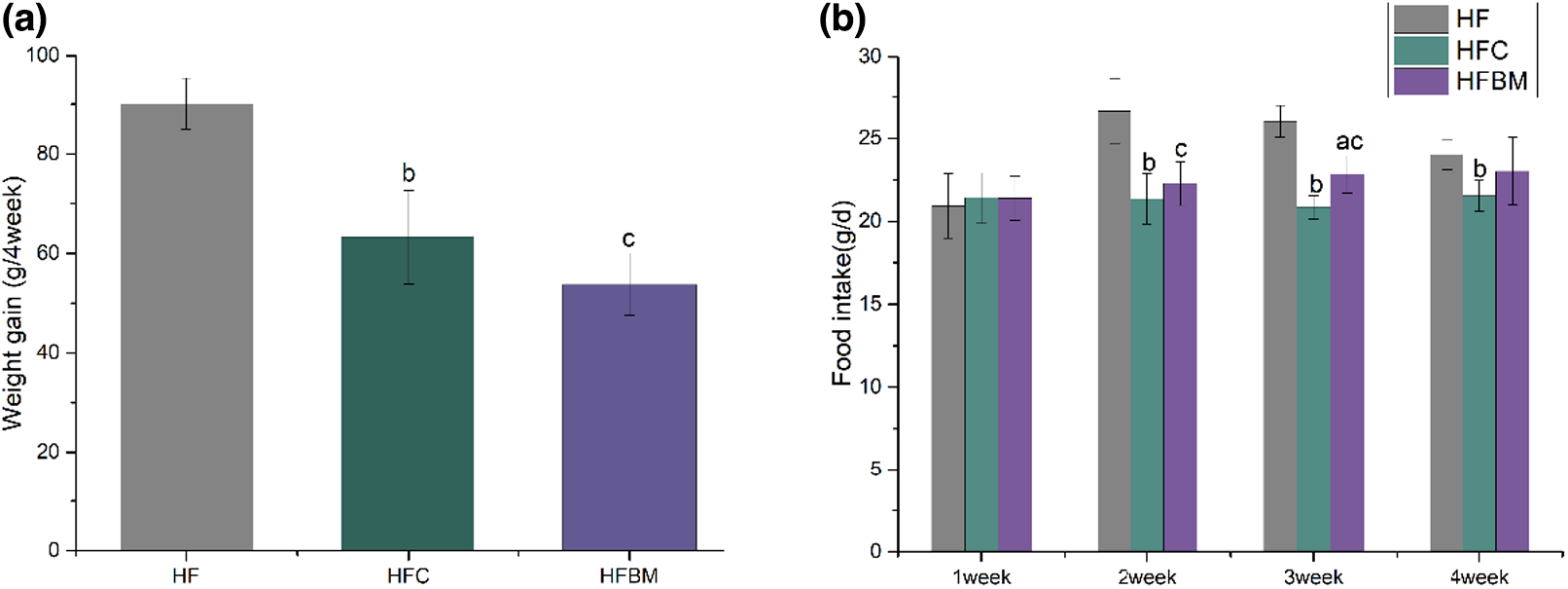
Effects of capsaicin plus dietary fibers on body weight (a) and food intake (B) of rats on a high‐fat diet. A, HFC group versus HFBM group; b, HF group versus HFC group; c, HF group versus HFBM group; (one‐way ANOVA followed by Dunnett's test; *p* < .05; *n* = 6). HF, high‐fat diet group; HFC, high‐fat diet group treated with CAP; HFBM, high‐fat diet group treated with CAP plus dietary fibers

Figure 1a shows the effect of CAP + DFs on the body weight of rats fed a high‐fat diet. The body weight in both the HFC and HFBM groups was significantly lower (−29.8% and −40.3%, respectively) than that in the HF group. There was no significant difference between the HFC and HFBM groups.

Figure 1b shows the effects of CAP + DFs on food intake at various stages in rats fed a high‐fat diet. In the first week, there were no differences in food intake among the HF, HFC, and HFBM groups. In the second week, the food intake of the HFC and HFBM groups was significant lower (−19.8% and −16.4%, respectively) than that of the HF group. In the third week, the food intake of the HFC and HFBM groups was significantly lower (−19.9% and −12.3%, respectively) than that of the HF group. Moreover, the HFBM group food intake was significantly higher (+14.5%) than that of the HFC group. In the fourth week, there was no significant difference in food intake in the HFBM group as compared with the HFC or HF groups, albeit that of the HFC group was lower than that of the HF group.

### 3.2 Effects of CAP + DFs on the blood biochemical indices of rats fed a high‐fat diet

Table 1 shows the effects of CAP + DFs on the blood biochemical indices of rats fed a high‐fat diet. Total cholesterol (TC) values in the HFBM and HFC groups were significantly lower (−49.8% and − 29.6%, respectively) than in the HF group. The TC value of the HFBM group was significantly higher than the HFC group's. Compared with the HF group, triglyceride (TG) levels were decreased in the HFC and HFBM groups (−34.2% and −32.9%, respectively). Low‐density lipoprotein cholesterol (LDL‐C) levels did not differ between the HFC and HF groups. Moreover, TG levels in the HFBM group were significantly lower than the HF group's. Compared with the HF group, high‐density lipoprotein cholesterol (HDL‐C) was significantly increased in the HFBM and HFC groups (+51.3% and +46.3%, respectively). With respect to the HF group, fasting plasma insulin and glucose levels, and HOMA‐IR values were all decreased in the HFC and HFBM groups.

**TABLE 1:** Effects of capsaicin combined with dietary fiber on blood biochemical indexes of high‐fat diet rats

Plasma parameters	HF	HFC	HFBM
TC (mmol/L)	4.32 ± 0.297	3.04 ± 0.212b	2.17 ± 0.269ac
TG (mmol/L)	0.76 ± 0.021	0.5 ± 0.014b	0.51 ± 0.004c
LDL‐C (mmol/L)	1.81 ± 0.028	1.52 ± 0.163	1.22 ± 0.219c
HDL‐C (mmol/L)	0.8 ± 0.014	1.17 ± 0.057b	1.21 ± 0.021c
Fasting insulin (mU/L)	5.65 ± 0.092	4.1 ± 0.198b	4.43 ± 0.318c
Fasting plasma glucose (mmol/L)	61.03 ± 1.018	48.26 ± 2.418b	47.89 ± 2.291c
HOMA‐IR	15.31 ± 0.505	8.78 ± 0.016b	9.40 ± 0.227c

*Note*: Values are the mean ± SD (*n* = 8). a, HFC group versus HFBM group; b, HFC group versus HF group; c, HF group versus HFBM group; (one‐way ANOVA followed by Dunnett's test, *p* < .05).

### 3.3 Effect of CAP + DFs on SCFA content in the cecum of rats fed a high‐fat diet

According to Table 2, the HFBM group exhibited most of the contents' changes, that is, acetic acid, +13.8% versus the HF group; n‐valeric acid increased in both the HFBM and HFC groups; i‐valeric acid, +25.1% versus the HF group; butyric acid increased versus the HF and HFC groups; total SCFAs, +45.8% versus the HF group. And, for the HFC group, total SCFAs increased by +29.3% versus the HF group. Finally, propionic acid levels were alike in the three groups.

**TABLE 2:** Effects of capsaicin combined with dietary fiber on cecal contents SCFA excretion of high‐fat diet rats

SCFAs	HF	HFC	HFBM
Acetic acid	13.48 ± 0.730	14.52 ± 0.557	15.35 ± 0.211c
Propionic acid	9.71 ± 0.286	10.15 ± 0.591	11.23 ± 1.017
butyric acid	5.22 ± 0.300	5.59 ± 0.219	8.11 ± 0.495ac
i‐butyric acid	7.72 ± 0.185	7.98 ± 0.018	8.26 ± 0.690
i‐valeric acid	4.54 ± 0.162	5.06 ± 0.080	5.68 ± 0.555c
n‐valeric acid	ND	4.39 ± 0.112	5.08 ± 0.517
Total	36.84 ± 5.96	47.65 ± 0.265b	53.71 ± 2.044c

*Note*: Values are the mean ± SD (*n* = 8). a, HFC group versus HFBM group; b, HFC group versus HF group; c, HF group versus HFBM group; (one‐way ANOVA followed by Dunnett's test, *p* < .05) ND: Not Detected.

### 3.4 Effects of CAP + DFs on gut microbiota 16 s RNAs of rats fed a high‐fat diet

**FIGURE 2:**
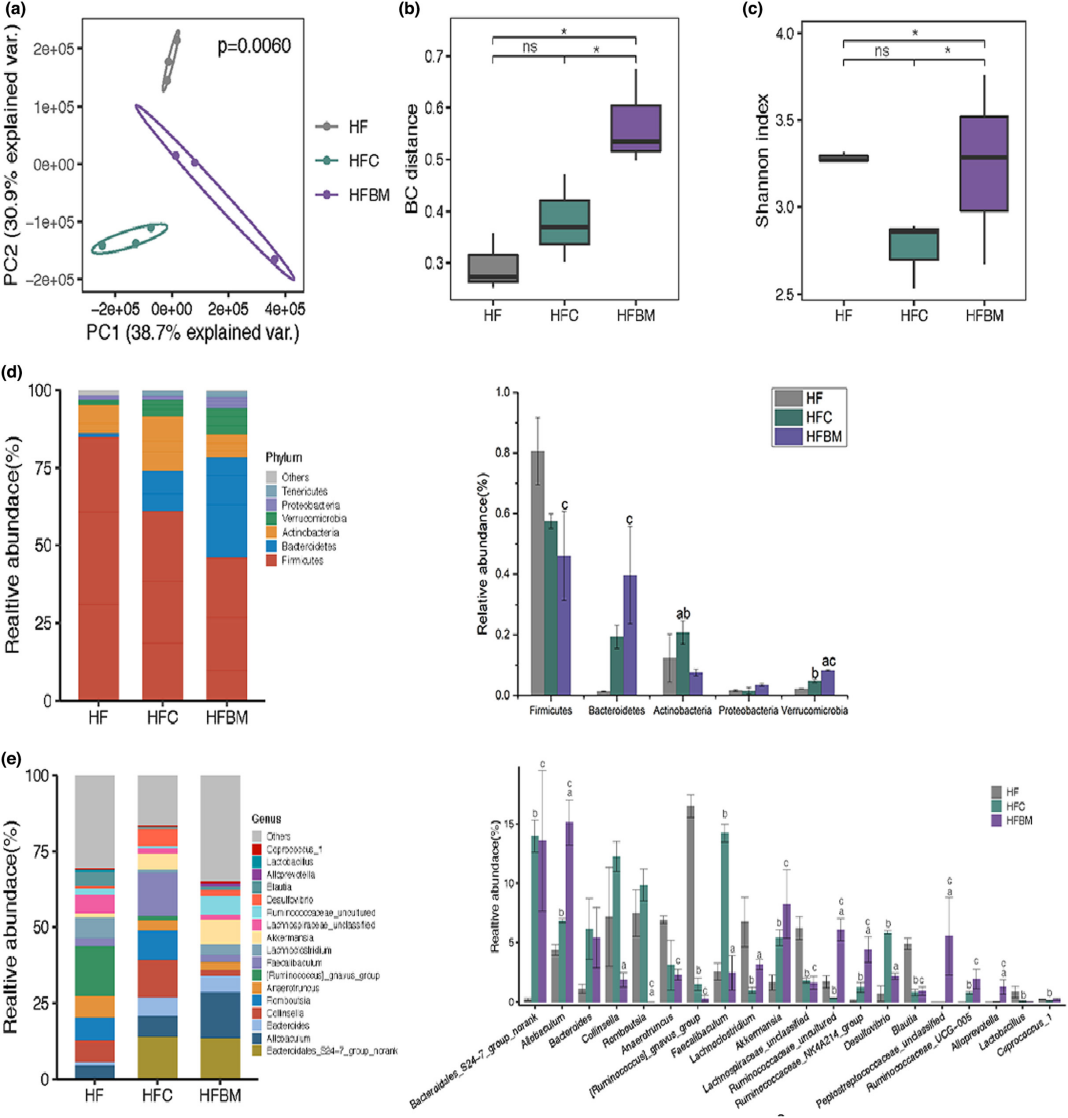
Effects of capsaicin plus dietary fibers on cecal gut microbiota of rats (a): Principal component analysis (PCA) at OTU level; (b): Shannon index at OTU level. (c): Bray–Curtis distance at OTU level. (d): Phylum‐level bar plot and differences in relative abundance of microorganisms at the phylum level. (e): Genus‐level bar plot and differences in relative abundance of the first 20 species of microorganisms. A, HFC group versus HFBM group; b, HF group versus HFC group; c, HF group versus HFBM group; (one‐way ANOVA followed by Dunnett's test; *p* < .05; *n* = 6). HF, high‐fat diet group; HFC, high‐fat diet group treated with CAP; HFBM, high‐fat diet group treated with CAP plus dietary fibers

Figure 2a shows the principal component analysis (PCA) of the intestinal microorganisms intersample diversity in rats fed a high‐fat diet added with CAP + DFs. The HFC and HFBM groups had different degrees of separation from the HF group, with those of the HFC group being more prominent.

Figure 2b shows the Shannon index analysis of α diversity among gut microbial samples from rats fed a high‐fat diet. Thus, in the HFBM group the α diversity was increased versus that of the HF and HFC groups. Conversely, in the HFC group the α diversity was slightly decreased versus the HF group.

Figure 2C shows the β diversity analysis using Bray–Curtis (BC) distance of gut microbial samples from rats fed a high‐fat diet. The β diversity of the HFBM group was increased versus those of the HF and HFC groups. However, the HFC group difference was not significantly higher than that of the HF group. Results of both α and β diversity analyses indicated that the gut floras of SD rats treated with CAP significantly changed. This was especially true with the CAP + DFs treatment, which resulted in significantly increased α and β diversities.

The gut flora compositions at the level of phylum are shown in Figure 2D. The five most abundant phyla were *Firmicutes*, *Verrucomicrobia*, *Bacteroidetes*, *Actinobacteria*, and *Proteobacteria*. Compared with the HF group, *Firmicutes* significantly decreased, while *Bacteroidetes* significantly increased, in the HFBM group. *Verrucomicrobia* significantly increased in both the HFC and HFBM groups.

Figure 2e shows the gut flora compositions at the genera level. The abundance of *Allobaculum* genus was significantly more represented in the HFBM group than in both the HFC and HF groups. The abundance of *Bacteroidales* S24‐7 genus was significantly higher in the HFC and HFBM groups than in the HF group. The *Akkermansia* genus abundance was higher in the HFC and HFBM groups than in the HF group—that of the HFBM group being slightly but not significantly higher than the HFC group's. The abundance of the *Blautia* genus was significantly higher in the HF group than in the HFC and HFBM groups. The *Desulfovibrio* genus abundance was significantly higher in the HFC group than in both the HF and HFBM groups. Finally, the *Desulfovibrio* abundance in the HFBM group was lesser than the HF group's.

### 3.5 Metabolomic analysis of cecal contents in rats fed a high‐fat diet added with CAP + DFs

**FIGURE 3:**
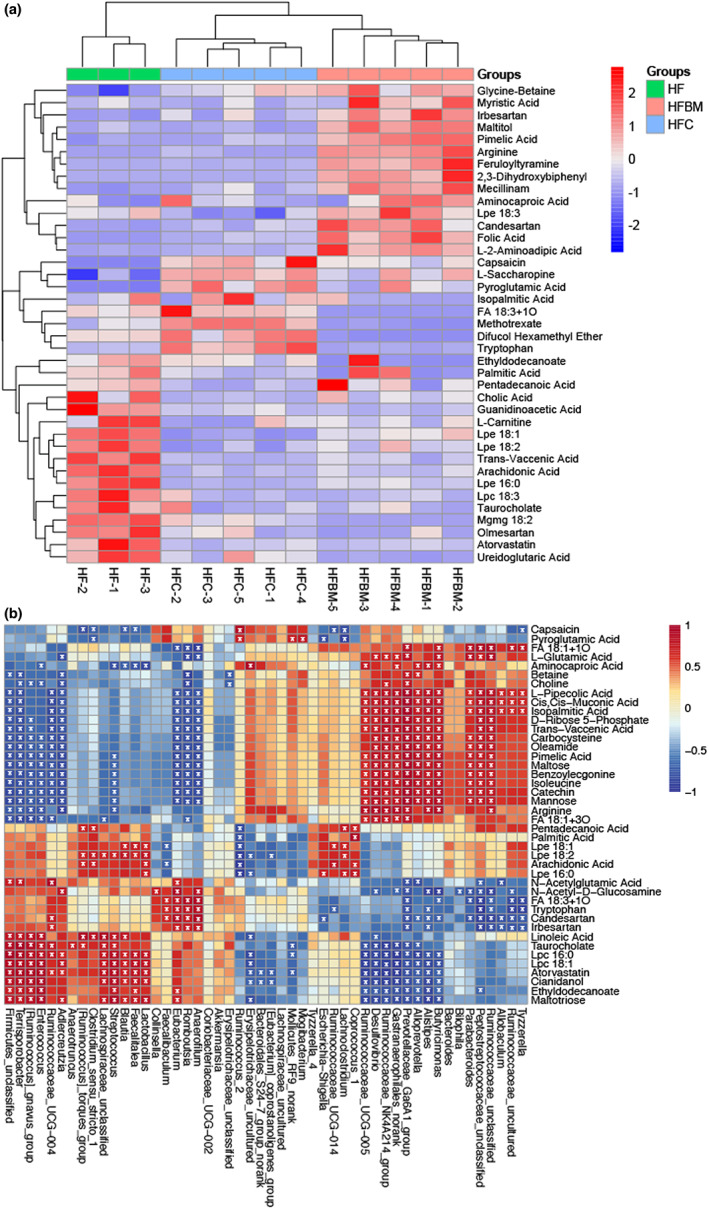
Effects of capsaicin plus dietary fibers on metabolites in the cecum. (a) Hierarchical clustering analysis of the significantly different metabolites based on an average clustering algorithm with Pearson distance. (b) Pearson correlation heatmap between key gut microbial taxa and profiles metabolites. Red color denotes a positive association; blue denotes a negative association; and white denotes no association. Data are expressed as mean values ± SDs (*n* = 5). One‐way ANOVA with Bonferroni post hoc test was used for statistical data analysis. *p < .05. HF, high‐fat diet group; HFC, high‐fat diet group treated with CAP; HFBM, high‐fat diet group treated with CAP plus dietary fibers

According to the comparison of the detected ion peaks and to the referenced database, a total of 661 compounds were detected in the cecal contents of each treatment group. Their Variable Importance for the Projection (VIP) values were calculated according to the OPLS‐DA model. When VIP >1, the difference reached a significant level. A total of 39 kinds of glucose and lipid metabolites were screened. Figure 3a shows the heatmap of hierarchical cluster analysis of such cecal metabolites. Collections of the same group of samples appeared in the same cluster, and the heatmap clearly distinguished the degrees of difference and similarity among metabolites. These results indicated that HFC, HFBM, and HF groups all belonged to different clusters.

Table 3 lists 18 substances related to lipid metabolism screened from 39 metabolites with VIP >1. As shown, arginine was significantly higher in the HFBM group than in both HF and HFC groups. Tryptophan was significantly increased in the HFBM and HFC groups. The Lpe:16:0, Lpe18:1, Lpe18:2, Lpe18:3, and Lpc18:3 in the HFC and HFBM groups were all significantly lower than in the HF group. Moreover, Lpe18:1 was higher in the HFBM group than in the HFC group. FA 18:3 + 1O was lower in the HFBM group than in the HF and HFC groups. Finally, Candesartan was significantly higher in the HFBM group than in both HF and HFC groups.

**TABLE 3:** Effects of capsaicin combined with dietary fiber on significantly different metabolites of cecum in rats

Alignment ID	Rt (min)	Metabolite name	Formula	Ontology	VIP	HF	HFC	HFBM
pos_00587	10.532	Arginine	C6H14N4O2	L‐alpha‐amino acids	1.10	5735.86 ± 4463.04	38,555.85 ± 52,829.28	652,664.23 ± 125,630.01ac
neg_13273	3.831	Atorvastatin	C33H35FN2O5	Diphenylpyrroles	1.14	120,485.66 ± 50,100.10	22,498.34 ± 10,121.04b	20,590.06 ± 6468.79c
neg_09735	5.62	Candesartan	C24H20N6O3	Biphenyls and derivatives	1.08	69,048.32 ± 7640.00	229,188.22 ± 106,904.06	1,000,802.85 ± 346,326.27ac
pos_02904	2.898	FA 18:3 + 1O	C18H30O3	Oxidized fatty acids	1.28	192,330.81 ± 34,915.24	303,215.62 ± 158,037.88	3434.43 ± 2423.08ac
neg_09356	6.878	Irbesartan	C25H28N6O	Biphenyls and derivatives	1.07	27,468.89 ± 5611.02	66,930.60 ± 38,060.57	187,368.19 ± 59,872.71ac
pos_00464	12.596	L‐Carnitine	C7H15NO3	Carnitines	1.05	269,632.71 ± 134,900.91	98,075.71 ± 66,713.48b	117,762.66 ± 37,813.58c
pos_10486	8.404	Lpc 18:3	C26H48NO7P	Lipids	1.03	11,467.34 ± 2730.94	3609.75 ± 1718.31b	3239.93 ± 818.90c
neg_10241	7.633	Lpe 16:0	C21H44NO7P	Lipids	1.08	758,101.52 ± 156,073.81	89,541.08 ± 24,341.41b	207,984.32 ± 54,531.13c
neg_11068	7.548	Lpe 18:1	C23H46NO7P	Lipids	1.27	186,457.92 ± 16,482.71	37,684.70 ± 9156.57b	95,095.45 ± 18,616.20ac
neg_11003	7.598	Lpe 18:2	C23H44NO7P	Lipids	1.25	72,295.74 ± 10,523.56	22,604.58 ± 7772.85b	35,467.47 ± 10,187.53c
pos_09144	7.655	Lpe 18:3	C23H42NO7P	Lipids	1.02	8270.85 ± 1238.08	5183.85 ± 1629.31b	11,182.34 ± 2301.70ac
neg_13388	0.691	Mgmg 18:2	C27H48O9	Lipids	1.35	15,082.73 ± 1399.59	4614.24 ± 2203.78b	360.49 ± 67.89ac
neg_01917	1.134	Myristic acid	C14H28O2	Long‐chain fatty acids	1.08	1534.017 ± 869.39	1069.46 ± 1018.17	5519.48 ± 3742.28ac
neg_02838	2.827	Palmitic acid	C16H32O2	Long‐chain fatty acids	1.17	32,840.95 ± 11,422.01	11,885.71 ± 4671.99	20,947.91 ± 26,117.09
neg_02311	2.845	Pentadecanoic acid	C15H30O2	Long‐chain fatty acids	1.17	33,051.42 ± 5407.58	9295.75 ± 4303.60	32,195.76 ± 32,118.96
neg_00547	7.85	Pimelic acid	C7H12O4	Medium‐chain fatty acids	1.18	28,115.04 ± 12,103.73	46,685.43 ± 13,976.33	187,274.16 ± 36,124.41ac
neg_03696	2.811	Trans‐vaccenic acid	C18H34O2	Long‐chain fatty acids	1.18	298,596.20 ± 54,509.63	21,362.99 ± 9355.81b	44,612.14 ± 26,576.22c
neg_01285	4.388	Tryptophan	C11H12N2O2	Indolyl carboxylic acids and derivatives	1.24	30,938.15 ± 2239.83	171,538.69 ± 51,119.81b	11,0447.47 ± 5043.84c

*Note*: Values are the mean ± SD (*n* = 8). a, HFC group versus HFBM group; b, HFC group versus HF group; c, HF group versus HFBM group; (one‐way ANOVA followed by Dunnett's test, *p* < .05).

Pearson correlation heatmap analysis showed that a link existed between gut microbiota genera and metabolites (Figure 3b). We found that *Bacteroidales* S24‐7 genus negatively correlated with Atorvastatin and Cianidanol; *Allobaculum* genus negatively correlated with N‐Acetyl‐D‐Glucosamine. Conversely, Candesartan and Irbesartan positively correlated with L‐Pipecolic acid and Cis‐Muconic acid; and *Collinsella* positively correlated with N‐Acetyl‐D‐Glucosamine.

## 4 DISCUSSION

CAP and DFs are two natural foods that regulate lipid metabolism abnormalities (Wang, Tang, et al., 2020; Zhang, Xiao, et al., 2020) In this study, CAP and DFs were added to the high‐fat diet of rats. After giving this diet for a set time period, the rats' blood composition, intestinal flora, and metabolites were analyzed. In both the HFBM and HFC groups the rats' body weights were lower (*p* < .05) than in the HF group. This indicated that CAP helped reduce body weight. From the second week onward, the food intake by the HFC and HFBM groups was lesser (*p* < .05) than that by the HF group, suggesting that CAP reduced rats' appetite. Our results are consistent with those of Wang et al. (Wang, Tang, et al., 2020) who found that CAP played a role in weight loss by activating genes, such as *PYY*, which suppressed appetite.

Generally, obesity somewhat correlates with lipid disorders. Four blood lipids—TC, TG, LDL‐C, and HDL‐C—are important indicators used to reveal changes in blood lipids (Chen et al., 2021). Earlier studies showed that HDL‐C levels negatively correlate with carotid atherosclerosis progression (Hedayatnia et al., 2020; Teis et al., 2021), while higher LDL‐C levels and lower HDL‐C levels might correlate with cardiovascular diseases (CVDs) such as stroke (Zhang, Wei, et al., 2020). This LDL‐C/HDL‐C ratio was proved to be more valuable than any single lipid component—especially LDL‐C—to predict CVDs risk (Hedayatnia et al., 2020; Zhang, Wei, et al., 2020).

Here, we analyzed the blood indices of rats fed a high‐fat diet. Our results indicated that there was no significant difference in LDL‐C levels in rats on a high‐fed diet added with CAP alone. Conversely, CAP + DFs significantly (*p* < .05) reduced blood LDL‐C levels. However, in the HFC and HFBM groups HDL‐C levels were significantly (*p* < .05) higher than in the HF group, revealing another effect of CAP alone or + DFs. Notably, a high‐fat diet increases TC and TG levels in the body. In our study, CAP decreased both TC and TG levels. This was especially true with the CAP + DFs, in which TC inhibition was associated with a better prognosis of an abnormal lipid profile than in the CAP alone group.

Insulin resistance related to glucose is a metabolic defect most importantly resulting in obesity (Bhattacharyya et al., 2019). Fasting plasma glucose and insulin levels are important parameters used to judge insulin resistance (Biesiekierski et al., 2019). Our present results showed that CAP, by itself or CAP + DFs, improved fasting plasma glucose and insulin levels, and the HOMA‐IR (*p* < .05). This revealed that CAP exerted a regulatory effect on insulin levels, consistent with the study of Zhang et al. (2017). However, the absence of any significant difference between the HFC and HFBM groups suggests that the antidiabetic effects observed resulted from CAP's effects.

The activity of gut microbes on undigested dietary fibers is the main source of the SCFAs, which primarily include acetic, propionic, and butyric acids. Collectively, SCFAs play a variety of roles that protect body homeostasis (Skaarud et al., 2021; Zhang, Dogan, et al., 2020). SCFAs' functional roles regulate the host's metabolism, immune system, and cell proliferation (Adebowale et al., 2019), and acts as an intestinal gluconeotroph improving glucose homeostasis and inhibiting hepatic lipid synthesis (De Vadder et al., 2014; Frost et al., 2014). In rodents, a large amount of acetic acid stimulates the hypothalamic center inhibiting appetite, thereby increasing energy consumption. Butyrate plays a key role in promoting colon health and possesses a remarkable variety of antineoplastic properties (Hernandez‐Maldonado et al., 2019). It can inhibit intestinal inflammation and regulate gut microbiota through the immune pathway. Moreover, 60–70% of the energy requirements for colon cell proliferation and differentiation can be met by butyrate (Gabbianelli et al., 2020; Vital et al., 2017).

In our study, acetic acid, butyric acid, and i‐valeric acid were significantly increased in rats fed CAP + DFs (*p* < .05), indicating that this treatment may promote energy consumption by regulating the host metabolism's and immune system, and by suppressing inflammation in the gut. This was especially true for CAP + DFs, which promoted the highest production of butyric acid. These results suggested that the CAP + DFs regimen more efficiently promoted the accumulation of SCFAs. This may be due to the fact that DFs could provide a greater number of digestive substrates to gut microbes, thereby promoting the DFs' conversion into SCFAs. We recall here that the accumulation of SCFAs is one of the mechanisms decreasing TC, TG, and LDL‐C levels and increasing HDL‐C levels (Fei et al., 2020; Li & Pan, 2020).

The intestinal microbiota composition of the rats fed CAP + DFs significantly differed from those of the other groups. CAP + DFs increased the diversity of the gut microbial community g. The Literature reports that the *Allobaculum* genus is a probiotic and belongs to the *Mycoplasma* family (Di et al., 2019). *Allobaculum* produces SCFAs (Li, Zhang, et al., 2019). Earlier studies showed that *Allobaculum* abundance is related to increases or decreases in body weight (Di et al., 2019). Compared with the HF group, gut abundance of *Allobaculum* genus was significantly (*p* < .05) high in rats fed either CAP + DFs or CAP alone. Moreover, *Allobaculum* abundance was greater with CAP + DFs than with CAP alone, indicating that the former treatment was more effective on weight loss. This view consists of the weight changes observed with the two treatments. Although the weight difference did not reach significance, the mean body weight of rats on a high‐fat diet added with CAP + DFs was slightly lower than that of rats on high‐fat diet plus CAP alone. The lack of statistical significance between the HFC and HFBM groups might have been due to the too brief duration of the feeding cycle, which did not affect enough the differences in body weight.

Being among the dominant bacteroid family members, the *Bacteroidales* S24‐7 genus is a strain of fermentation bacteria that degrades carbohydrates into SCFAs (Gao et al., 2020; Monk et al., 2017). It usually degrades complex polysaccharides into acetic, propionic, and succinic acids (Ormerod et al., 2016). In this study, either CAP + DFs or CAP alone significantly (*p* < .05) increased the gut's abundance of the *Bacteroidales* S24‐7 strain in rats fed a high‐fat diet. This might be one of the reasons why the accumulation of short‐chain fatty acids was more abundant. The results of this study are in line with previous ones, which found that the abundance of the intestinal *Bacteroidales* S24‐7 genus was improved by CAP gavage in rats fed a high‐fat diet (Wang, Tang, et al., 2020).

The bacterial *Akkermansia* genus, a novel strain of probiotics, resides in the gut and exerts lipid‐lowering effects. Previous studies showed that *Akkermansia'*s abundance negatively correlates with some metabolic disorders of humans and mice such as inflammatory bowel disease, obesity, autism, and type 2 diabetes, etc. (Meng et al., 2020). Because of this, it was important exploring CAP's effects on an intestinal microbiome member like *Akkermansia* (Shen et al., 2017; Wang, Tang, et al., 2020). Our results showed that adding CAP + DFs or CAP alone to a high‐fat diet significantly (*p* < .05) increased versus the HF group the rats' gut abundance of *Akkermansia*. This effect was more intense in the HFBM than in the HFC group indicating that CAP + DFs may achieve better lipid‐lowering effects.

*Desulfovibrio* is a genus of sulfate‐reducing bacteria that stimulates the gut immune response promoting an inflammation that in severe cases can lead to colon cancer (Figliuolo et al., 2017; Grubb et al., 2020). The abundance of *Desulfovibrio* genus in the HFBM group was significantly (*p* < .05) lower than in the HFC group, indicating that adding CAP + DFs effectively reduced the abundance of the harmful *Desulfovibrio* bacteria, thereby reducing colitis and the risk of colon cancer.

Metabolomics are a group of scientific methods aimed at disclosing all the biological fingerprints associated with metabolites, molecular intermediates, and products of metabolism (Li, Qin, et al., 2019). Metabolic profiling provides a direct, instantaneous snapshot of the physiology of the targeted cell and organism (Kalim & Rhee, 2017). Using metabolites' analysis, we observed a series of change in responsive metabolites and screened out 18 glucose and lipid metabolites. We found that CAP + DFs increased (*p* < .05) the levels of arginine, Candesartan, and Irbesartan in cecum. Arginine plays a key role in the treatment of intestinal inflammation, improving gut microbiome, and reducing both oxidative stress and neutrophil infiltration (Bowerman et al., 2020; Zhang & Li, 2014). Collectively, our data indicated that CAP + DFs could block gut inflammation and improve gut microbiome. Candesartan—an AT1 receptor antagonist—is widely used to treat hypertension and CVDs (Garcia‐Garrote et al., 2019; Poudel & Kim, 2021). Irbesartan is an angiotensin receptor blocker (ARB) that at high doses effectively lowers blood pressure, mitigates carotid artery atherosclerosis, and relieves clinical symptoms (Li & Yao, 2018). The level of candesartan and irbesartan were increased in the cecum of rats fed CAP + DFs suggest that this regimen may prevent hypertension.

Tryptophan is an essential amino acid owning an indole ring that is present in dietary proteins (Kałużna‐Czaplińska et al., 2019). Although tryptophan is the least abundant amino acid in proteins and hence in cells, it is a precursor of the biosynthesis of a large number of microbial metabolites. In vitro studies showed that some patients produced, transported, and catabolized lesser amounts of tryptophan. This indicated that tryptophan plays a crucial role in human T cell differentiation, immune regulation, and neurological function (Sandgren & Brummer, 2018). Tryptophan's levels in the cecal contents of rats fed CAP alone or CAP + DFs were significantly (*p* < .05) increased, suggesting that either treatment may improve overall immune function.

PC, LPE, and LPC are involved in glycerophospholipid metabolism (Zhou et al., 2020). In our research, Lpe:16:0, Lpe18:1, Lpe18:2, Lpe18:3, and Lpc18:3 were all significantly (*p* < .05) decreased in rats fed CAP alone or CAP + DFs. Interestingly, when compared with CAP alone, both Lpe18:1 and Lpc18:3 levels were significantly (*p* < .05) higher in rats fed CAP + DFs. This result has been due to the increased variety of intestinal flora, which promotes the maintenance of intestinal phospholipid levels by some nonpathogenic bacteria (Zou et al., 2020).

## 5 CONCLUSIONS

There are large numbers of complex microbial communities in the human gut. The interaction between gut microbes and the host forms a network system that maintains bodily health. We studied the effects of adding CAP + DFs on the intestinal microbiome and metabolism on rats kept on a high‐fat diet. Our results showed that CAP + DFs added to a high‐fat diet (1) regulated rats' lipid metabolism better than CAP alone did; (2) improved the diversity of intestinal microbiome, optimizing its composition, while reducing the abundance of *Desulfovibrio* genus bacteria in rats fed a high‐fat diet; and (3) promoted the accumulation of butyric acid resulting in increases in total SCFAs.

## CONFLICT OF INTEREST

All authors declare that they have no conflict of interest. They also declare that have no financial and personal relationships with other people or organizations that can inappropriately influence their work; that there is no professional or other personal interest of any nature or kind in any product, service, and/or company that could be construed as influencing the data presented in, or the review of, the manuscript entitled, “Capsaicin combined with dietary fiber prevents high‐fat diet‐associated aberrant lipid metabolism by improving the structure of intestinal flora.”

## ETHICS STATEMENT

All procedures followed were in accordance with the ethical standards of the responsible Committees on Human Experimentation, Institutional Animal Care, and Use Committee of Southwest University.

## Supporting information


Figure S1

## Data Availability

The data that support the findings of this study are available on request from the corresponding author.

## References

[fsn33043-bib-0001] Adebowale, T. O. , Yao, K. , & Oso, A. O. (2019). Major cereal carbohydrates in relation to intestinal health of monogastric animals: A review. Animal Nutrition, 5(4), 331–339. 10.1016/j.aninu.2019.09.001 31890909 PMC6920401

[fsn33043-bib-0002] Afrose, S. , Hossain, M. S. , Maki, T. , & Tsujii, H. (2009). Karaya root saponin exerts a hypocholesterolemic response in rats fed a high‐cholesterol diet. Nutrition Research, 29(5), 350–354. 19555817 10.1016/j.nutres.2009.05.008

[fsn33043-bib-0003] Barber, T. M. , Kabisch, S. , Pfeiffer, A. E. H. , & Weickert, M. O. (2020). The health benefits of dietary fibre. Nutrients, 12(10), 3209. 10.3390/nu12103209 33096647 PMC7589116

[fsn33043-bib-0004] Bhattacharyya, S. , Feferman, L. , & Tobacman, J. K. (2019). Distinct effects of carrageenan and high‐fat consumption on the mechanisms of insulin resistance in nonobese and obese models of type 2 diabetes. Journal of Diabetes Research, 2019, 1–14. 10.1155/2019/9582714 PMC6501429 31179345

[fsn33043-bib-0005] Biesiekierski, J. R. , Livingstone, K. M. , & Moschonis, G. (2019). Personalised nutrition: Updates, gaps and next steps. Nutrients, 11(8), 1793. 10.3390/nu11081793 31382527 PMC6722533

[fsn33043-bib-0006] Bowerman, K. L. , Rehman, S. F. , Vaughan, A. , Lachner, N. , Budden, K. F. , Kim, R. Y. , Wood, D. L. A. , Gellatly, S. L. , Shukla, S. D. , Wood, L. G. , Yang, I. A. , Wark, P. A. , Hugenholtz, P. , & Hansbro, P. M. (2020). Disease‐associated gut microbiome and metabolome changes in patients with chronic obstructive pulmonary disease. Nature Communications, 11(1), 5886. 10.1038/s41467-020-19701-0 PMC7676259 33208745

[fsn33043-bib-0007] Chen, D.‐Y. , Sawamura, T. , Dixon, R. A. F. , Sanchez‐Quesada, J. L. , & Chen, C.‐H. (2021). Autoimmune rheumatic diseases: An update on the role of atherogenic electronegative LDL and potential therapeutic strategies. Journal of Clinical Medicine, 10(9), 1992. 10.3390/jcm10091992 34066436 PMC8124242

[fsn33043-bib-0008] Chen, M.‐Y. , Qiu, Z.‐W. , Tang, H.‐M. , Zhuang, K.‐H. , Cai, Q.‐Q. , Chen, X.‐l. , & Li, H.‐B. (2019). Efficacy and safety of bifid triple viable plus aminosalicylic acid for the treatment of ulcerative colitis a systematic review and meta‐analysis. Medicine, 98(47), e17955. 10.1097/md.0000000000017955 31764796 PMC6882635

[fsn33043-bib-0009] Cheng, X. , Zheng, J. , Lin, A. , Xia, H. , Zhang, Z. , Gao, Q. , Lv, W. , & Liu, H. (2020). A review: Roles of carbohydrates in human diseases through regulation of imbalanced intestinal microbiota. Journal of Functional Foods, 74, 104197. 10.1016/j.jff.2020.104197

[fsn33043-bib-0010] De Vadder, F. , Kovatcheva‐Datchary, P. , Goncalves, D. , Vinera, J. , Zitoun, C. , Duchampt, A. , Bäckhed, F. , & Mithieux, G. (2014). Microbiota‐generated metabolites promote metabolic benefits via gut‐brain neural circuits. Cell, 156(1–2), 84–96. 10.1016/j.cell.2013.12.016 24412651

[fsn33043-bib-0011] Di, S. , Wang, Y. , Han, L. , Bao, Q. , Gao, Z. , Wang, Q. , Yang, Y. , Zhao, L. , & Tong, X. (2019). The intervention effect of traditional Chinese medicine on the intestinal Flora and its metabolites in glycolipid metabolic disorders. Evidence‐based Complementary and Alternative Medicine, 2019, 1–13. 10.1155/2019/2958920 PMC6582858 31275408

[fsn33043-bib-0012] Farzi, A. , Ip, C. K. , Reed, F. , Enriquez, R. , Zenz, G. , Durdevic, M. , Zhang, L. , Holzer, P. , & Herzog, H. (2021). Lack of peptide YY signaling in mice disturbs gut microbiome composition in response to high‐fat diet. FASEB Journal, 35(4), e21435. 10.1096/fj.202002215R 33749879 PMC8251710

[fsn33043-bib-0013] Fei, Y. , Wang, Y. , Pang, Y. , Wang, W. , Zhu, D. , Xie, M. , Lan, S. , & Wang, Z. (2020). Xylooligosaccharide modulates gut microbiota and alleviates colonic inflammation caused by high fat diet induced obesity. Frontiers in Physiology, 10, 1601. 10.3389/fphys.2019.01601 32038285 PMC6987399

[fsn33043-bib-0014] Figliuolo, V. R. , dos Santos, L. M. , Abalo, A. , Nanini, H. , Santos, A. , Brittes, N. M. , Bernardazzi, C. , de Souza, H. S. P. , Vieira, L. Q. , Coutinho‐Silva, R. , & CMLM, C. (2017). Sulfate‐reducing bacteria stimulate gut immune responses and contribute to inflammation in experimental colitis. Life Sciences, 189, 29–38. 10.1016/j.lfs.2017.09.014 28912045

[fsn33043-bib-0015] Frost, G. , Sleeth, M. L. , Sahuri‐Arisoylu, M. , Lizarbe, B. , Cerdan, S. , Brody, L. , Anastasovska, J. , Ghourab, S. , Hankir, M. , Zhang, S. , Carling, D. , Swann, J. R. , Gibson, G. , Viardot, A. , Morrison, D. , Louise Thomas, E. , & Bell, J. D. (2014). The short‐chain fatty acid acetate reduces appetite via a central homeostatic mechanism. Nature Communications, 5, 3611. 10.1038/ncomms4611 PMC4015327 24781306

[fsn33043-bib-0016] Gabbianelli, R. , Bordoni, L. , Morano, S. , Calleja‐Agius, J. , & Lalor, J. G. (2020). Nutri‐epigenetics and gut microbiota: How birth care, bonding and breastfeeding can influence and be influenced? International Journal of Molecular Sciences, 21(14), 5032. 10.3390/ijms21145032 32708742 PMC7404045

[fsn33043-bib-0017] Gao, Y. , Yang, L. , Chin, Y. , Liu, F. , Li, R. W. , Yuan, S. , Xue, C. , Xu, J. , & Tang, Q. (2020). Astaxanthin n‐octanoic acid diester ameliorates insulin resistance and modulates gut microbiota in high‐fat and high‐sucrose diet‐fed mice. International Journal of Molecular Sciences, 21(6), 2149. 10.3390/ijms21062149 32245087 PMC7139465

[fsn33043-bib-0018] Garcia‐Garrote, M. , Perez‐Villalba, A. , Garrido‐Gil, P. , Belenguer, G. , Parga, J. A. , Perez‐Sanchez, F. , Labandeira‐Garcia, J. L. , Fariñas, I. , & Rodriguez‐Pallares, J. (2019). Interaction between angiotensin type 1, type 2, and mas receptors to regulate adult neurogenesis in the brain ventricular‐subventricular zone. Cell, 8(12), 1551. 10.3390/cells8121551 PMC6952803 31801296

[fsn33043-bib-0019] Gong, T. , Wang, H. , Liu, S. , Zhang, M. , Xie, Y. , & Liu, X. (2022). Capsaicin regulates lipid metabolism through modulation of bile acid/gut microbiota metabolism in high‐fat‐fed SD rats. Food & Nutrition Research, 66, 8289. 10.29219/fnr.v66.8289 PMC9180124 35721805

[fsn33043-bib-0020] Grubb, D. S. , Wrigley, S. D. , Freedman, K. E. , Wei, Y. , Vazquez, A. R. , Trotter, R. E. , Wallace, T. C. , Johnson, S. A. , & Weir, T. L. (2020). HAGE‐2 study: Supplemental bacteriophages extend *Bifidobacterium animalis* subsp. *lactis* BL04 benefits on gut health and microbiota in healthy adults. Nutrients, 12(8), 2474. 10.3390/nu12082474 32824480 PMC7468981

[fsn33043-bib-0021] Hedayatnia, M. , Asadi, Z. , Zare‐Feyzabadi, R. , Yaghooti‐Khorasani, M. , Ghazizadeh, H. , Ghaffarian‐Zirak, R. , Nosrati‐Tirkani, A. , Mohammadi‐Bajgiran, M. , Rohban, M. , Sadabadi, F. , Rahimi, H. R. , Ghalandari, M. , Ghaffari, M. S. , Yousefi, A. , Pouresmaeili, E. , Besharatlou, M. R. , Moohebati, M. , Ferns, G. A. , Esmaily, H. , & Ghayour‐Mobarhan, M. (2020). Dyslipidemia and cardiovascular disease risk among the MASHAD study population. Lipids in Health and Disease, 19(1), 42. 10.1186/s12944-020-01204-y 32178672 PMC7075010

[fsn33043-bib-0022] Hernandez‐Maldonado, L. M. , Blancas‐Benitez, F. J. , Zamora‐Gasga, V. M. , Cardenas‐Castro, A. P. , Tovar, J. , & Sayago‐Ayerdi, S. G. (2019). In vitro gastrointestinal digestion and colonic fermentation of high dietary fiber and antioxidant‐rich mango (*Mangifera indica* L.) “Ataulfo”‐based fruit bars. Nutrients, 11(7), 1564. 10.3390/nu11071564 31336740 PMC6682962

[fsn33043-bib-0023] Hosoi, T. , Kohda, T. , Matsuzaki, S. , Ishiguchi, M. , Kuwamura, A. , Akita, T. , Tanaka, J. , & Ozawa, K. (2016). Key role of heat shock protein 90 in leptin‐induced STAT3 activation and feeding regulation. British Journal of Pharmacology, 173(15), 2434–2445. 10.1111/bph.13520 27205876 PMC4945768

[fsn33043-bib-0024] Huang, C.‐N. , Wang, C.‐J. , Lin, C.‐L. , Lin, H.‐T. , & Peng, C.‐H. (2017). The nutraceutical benefits of subfractions of Abelmoschus esculentus in treating type 2 diabetes mellitus. PLoS One, 12(12), e0189065. 10.1371/journal.pone.0189065 29216237 PMC5720626

[fsn33043-bib-0025] Hui, S. , Liu, Y. , Chen, M. , Wang, X. , Lang, H. , Zhou, M. , Yi, L. , & Mi, M. (2019). Capsaicin improves glucose tolerance and insulin sensitivity through modulation of the gut microbiota‐bile acid‐FXR Axis in type 2 diabetic db/db mice. Molecular Nutrition & Food Research, 63(23), e1900608. 10.1002/mnfr.201900608 31539192

[fsn33043-bib-0026] Kalim, S. , & Rhee, E. P. (2017). An overview of renal metabolomics. Kidney International, 91(1), 61–69. 10.1016/j.kint.2016.08.021 27692817 PMC5380230

[fsn33043-bib-0027] Kałużna‐Czaplińska, J. , Gątarek, P. , Chirumbolo, S. , Chartrand, M. S. , & Bjørklund, G. (2019). How important is tryptophan in human health? Critical Reviews in Food Science and Nutrition, 59(1), 72–88. 28799778 10.1080/10408398.2017.1357534

[fsn33043-bib-0028] Kørner, C. J. , Du, X. , Vollmer, M. E. , & Pajerowska‐Mukhtar, K. M. (2015). Endoplasmic reticulum stress signaling in plant immunity—At the crossroad of life and death. International Journal of Molecular Sciences, 16(11), 26582–26598. 26556351 10.3390/ijms161125964 PMC4661823

[fsn33043-bib-0029] Li, Q. , & Pan, Y. (2020). Differential responses to dietary protein and carbohydrate ratio on gut microbiome in obese vs Lean Cats. Frontiers in Microbiology, 11, 591462. 10.3389/fmicb.2020.591462 33178173 PMC7596662

[fsn33043-bib-0030] Li, R. , Qin, X. , Liang, X. , Liu, M. , & Zhang, X. (2019). Lipidomic characteristics and clinical findings of epileptic patients treated with valproic acid. Journal of Cellular and Molecular Medicine, 23(9), 6017–6023. 10.1111/jcmm.14464 31162795 PMC6714506

[fsn33043-bib-0031] Li, T. , & Yao, W. (2018). Therapeutic effect of irbesartan combined with atorvastatin calcium in the treatment of rats with coronary heart disease. Experimental and Therapeutic Medicine, 16(5), 4119–4123. 10.3892/etm.2018.6669 30402154 PMC6200972

[fsn33043-bib-0032] Li, X.‐L. , Zhang, B. , Sun, M.‐J. , Bao, C.‐C. , Yuan, B.‐Y. , Xie, Q.‐F. , Wang, L.‐J. , & Wang, M.‐X. (2019). Mechanism of gut microbiota and Axl/SOCS3 in experimental autoimmune encephalomyelitis. Bioscience Reports, 39, BSR20190228. 10.1042/bsr20190228 31221818 PMC6603274

[fsn33043-bib-0033] Lin, P.‐H. , Chang, C.‐C. , Wu, K.‐H. , Shih, C.‐K. , Chiang, W. , Chen, H.‐Y. , Shih, Y. H. , Wang, K. L. , Hong, Y. H. , Shieh, T. M. , & Hsia, S.‐M. (2019). Dietary Glycotoxins, advanced glycation end products, inhibit cell proliferation and progesterone secretion in ovarian granulosa cells and mimic PCOS‐like symptoms. Biomolecules, 9(8), 327. 10.3390/biom9080327 31370285 PMC6723748

[fsn33043-bib-0034] Lindroos, A. K. , Moraeus, L. , Sipinen, J. P. , Lemming, E. W. , & Patterson, E. (2021). The contribution of foods and beverages of low nutritional value to the diets of Swedish adolescents, by food group, time and place. A nationally representative study. Nutrients, 13(7), 2450. 10.3390/nu13072450 34371960 PMC8308806

[fsn33043-bib-0035] Meng, X. , Wang, W. , Lan, T. , Yang, W. , Yu, D. , Fang, X. , & Wu, H. (2020). A purified aspartic protease from Akkermansia Muciniphila plays an important role in degrading Muc2. International Journal of Molecular Sciences, 21(1), 72. 10.3390/ijms21010072 PMC6982040 31861919

[fsn33043-bib-0036] Monk, J. M. , Lepp, D. , Wu, W. , Pauls, K. P. , Robinson, L. E. , & Power, K. A. (2017). Navy and black bean supplementation primes the colonic mucosal microenvironment to improve gut health. Journal of Nutritional Biochemistry, 49, 89–100. 10.1016/j.jnutbio.2017.08.002 28915390

[fsn33043-bib-0037] Ormerod, K. L. , Wood, D. L. A. , Lachner, N. , Gellatly, S. L. , Daly, J. N. , Parsons, J. D. , Dal'Molin, C. G. , Palfreyman, R. W. , Nielsen, L. K. , Cooper, M. A. , Morrison, M. , Hansbro, P. M. , & Hugenholtz, P. (2016). Genomic characterization of the uncultured Bacteroidales family S24‐7 inhabiting the guts of homeothermic animals. Microbiome, 4, 36. 10.1186/s40168-016-0181-2 27388460 PMC4936053

[fsn33043-bib-0038] Poudel, S. , & Kim, D. W. (2021). Developing pH‐modulated spray dried amorphous solid dispersion of candesartan Cilexetil with enhanced in vitro and in vivo performance. Pharmaceutics, 13(4), 497. 10.3390/pharmaceutics13040497 33917403 PMC8067465

[fsn33043-bib-0039] Rosca, A. E. , Iesanu, M. I. , Zahiu, C. D. M. , Voiculescu, S. E. , Paslaru, A. C. , & Zagrean, A.‐M. (2020). Capsaicin and gut microbiota in health and disease. Molecules, 25(23), 5681. 10.3390/molecules25235681 33276488 PMC7730216

[fsn33043-bib-0040] Sandgren, A. M. , & Brummer, R. J. M. (2018). ADHD‐originating in the gut? The emergence of a new explanatory model. Medical Hypotheses, 120, 135–145. 10.1016/j.mehy.2018.08.022 30220333

[fsn33043-bib-0041] Shen, W. , Shen, M. , Zhao, X. , Zhu, H. , Yang, Y. , Lu, S. , Tan, Y. , Li, G. , Li, M. , Wang, J. , Hu, F. , & le, S. (2017). Anti‐obesity effect of capsaicin in mice fed with high‐fat diet is associated with an increase in population of the gut bacterium Akkermansia muciniphila. Frontiers in Microbiology, 8, 1–10. 10.3389/fmicb.2017.00272 28280490 PMC5322252

[fsn33043-bib-0042] Skaarud, K. J. , Hov, J. R. , Hansen, S. H. , Kummen, M. , Valeur, J. , Seljeflot, I. , Bye, A. , Paulsen, V. , Lundin, K. E. A. , Trøseid, M. , Tjønnfjord, G. E. , & Iversen, P. O. (2021). Mortality and microbial diversity after allogeneic hematopoietic stem cell transplantation: Secondary analysis of a randomized nutritional intervention trial. Scientific Reports, 11(1), 11593. 10.1038/s41598-021-90976-z 34078971 PMC8172574

[fsn33043-bib-0043] Teis, A. , Cediel, G. , Amigó, N. , Julve, J. , Aranyó, J. , Andrés‐Cordón, J. , Puig‐Jové, C. , Castelblanco, E. , Gual‐Capllonch, F. , Ferrer‐Sistach, E. , Vallejo, N. , Juncà, G. , López‐Ayerbe, J. , de Antonio, M. , Domingo, M. , Santiago‐Vacas, E. , Codina, P. , Mauricio, D. , Lupón, J. , … Bayes‐Genis, A. (2021). Particle size and cholesterol content of circulating HDL correlate with cardiovascular death in chronic heart failure. Scientific Reports, 11(1), 3141. 10.1038/s41598-021-82861-6 33542459 PMC7862293

[fsn33043-bib-0044] Tian, X. , & Wang, H. (2019). The impact of having one parent absent on Children' food consumption and nutrition in China. Nutrients, 11(12), 3077. 10.3390/nu11123077 31861207 PMC6950458

[fsn33043-bib-0045] Vital, M. , Karch, A. , & Pieper, D. H. (2017). Colonic butyrate‐producing communities in humans: An overview using omics data. Msystems, 2(6), e00130‐17. 10.1128/mSystems.00130-17 29238752 PMC5715108

[fsn33043-bib-0046] Wang, H. , Lu, Y. , Yan, Y. , Tian, S. , Zheng, D. , Leng, D. , Wang, C. , Jiao, J. , Wang, Z. , & Bai, Y. (2020). Promising treatment for type 2 diabetes: Fecal microbiota transplantation reverses insulin resistance and impaired islets. Frontiers in Cellular and Infection Microbiology, 9, 455. 10.3389/fcimb.2019.00455 32010641 PMC6979041

[fsn33043-bib-0047] Wang, Y. , Tang, C. , Tang, Y. , Yin, H. , & Liu, X. (2020). Capsaicin has an anti‐obesity effect through alterations in gut microbiota populations and short‐chain fatty acid concentrations. Food & Nutrition Research, 64, 3525. 10.29219/fnr.v64.3525 PMC7054644 32180694

[fsn33043-bib-0048] Zhang, H. , Bai, Y. , Gao, M. , Zhang, J. , Dong, G. , Yan, F. , Ma, Q. , Fu, X. , Zhang, Q. , Li, C. , Shi, H. , Ning, Z. , Dai, J. , Li, Z. , Ming, J. , Xue, Q. , Si, C. , & Xiong, H. (2019). Hepatoprotective effect of capsaicin against concanavalin A‐induced hepatic injury via inhibiting oxidative stress and inflammation. American Journal of Translational Research, 11(5), 3029–3038. 31217872 PMC6556673

[fsn33043-bib-0049] Zhang, L. (2013). Research on Hypocholesterolemic effects and mechanism of Capsaicinoids on cholesterol metabolic in vivo and Viro. Southwest University.

[fsn33043-bib-0050] Zhang, Q. , Xiao, X. , Zheng, J. , Li, M. , Yu, M. , Ping, F. , Wang, T. , & Wang, X. (2020). Maternal inulin supplementation alters hepatic DNA methylation profile and improves glucose metabolism in offspring mice. Frontiers in Physiology, 11, 70. 10.3389/fphys.2020.00070 32116778 PMC7020697

[fsn33043-bib-0051] Zhang, S. , Dogan, B. , Guo, C. , Herlekar, D. , Stewart, K. , Scherl, E. J. , & Simpson, K. W. (2020). Short chain fatty acids modulate the growth and virulence of Pathosymbiont Escherichia coli and host response. Antibiotics‐Basel, 9(8), 462. 10.3390/antibiotics9080462 32751519 PMC7460008

[fsn33043-bib-0052] Zhang, S. , Ma, X. , Zhang, L. , Sun, H. , & Liu, X. (2017). Capsaicin reduces blood glucose by increasing insulin levels and glycogen content better than Capsiate in streptozotocin‐induced diabetic rats. Journal of Agricultural and Food Chemistry, 65(11), 2323–2330. 10.1021/acs.jafc.7b00132 28230360

[fsn33043-bib-0053] Zhang, X.‐X. , Wei, M. , Shang, L.‐X. , Lu, Y.‐M. , Zhang, L. , Li, Y.‐D. , Zhang, J. H. , Xing, Q. , Tu‐Erhong, Z. K. , Tang, B. P. , & Zhou, X.‐H. (2020). LDL‐C/HDL‐C is associated with ischaemic stroke in patients with non‐valvular atrial fibrillation: A case‐control study. Lipids in Health and Disease, 19(1), 217. 10.1186/s12944-020-01392-7 33028331 PMC7542146

[fsn33043-bib-0054] Zhang, Y.‐Z. , & Li, Y.‐Y. (2014). Inflammatory bowel disease: Pathogenesis. World Journal of Gastroenterology, 20(1), 91–99. 10.3748/wjg.v20.i1.91 24415861 PMC3886036

[fsn33043-bib-0055] Zhou, C. , He, M. , Peng, C. , Yu, J. , Li, Z. , Zhou, M. , Li, Y. , Yang, S. , Ouyang, H. , & Feng, Y. (2020). Pharmacokinetic and lipidomic assessment of the in vivo effects of Parishin A‐Isorhynchophylline in rat migraine models. Journal of Analytical Methods in Chemistry, 2020, 1–11. 10.1155/2020/9101598 PMC7362284 32695549

[fsn33043-bib-0056] Zou, D. Y. , Pei, J. W. , Lan, J. F. , Sang, H. , Chen, H. J. , Yuan, H. L. , Wu, D. , Zhang, Y. , Wang, Y. , Wang, D. , Zou, Y. , Chen, D. , Ren, J. , Gao, X. , & Lin, Z. Y. (2020). A SNP of bacterial blc disturbs gut lysophospholipid homeostasis and induces inflammation through epithelial barrier disruption. eBioMedicine, 52, 102652. 10.1016/j.ebiom.2020.102652 32058942 PMC7026729

