# Supplementary material for: Capsaicin combined with dietary fiber prevents high‐fat diet associated aberrant lipid metabolism by improving the structure of intestinal flora
Source: Food Sci Nutr. 2022 Sep 20;11(1):114–25. doi: 10.1002/fsn3.3043 (PMC9834886; doi:10.1002/fsn3.3043)
Supplement: Supplementary file 1 — Figure S1 [file FSN3-11-114-s001.docx]

**Figure Captions**

**Fig. S1.** Gas chromatogram of short-chain fatty acids in standard solution (20 mmol/L).


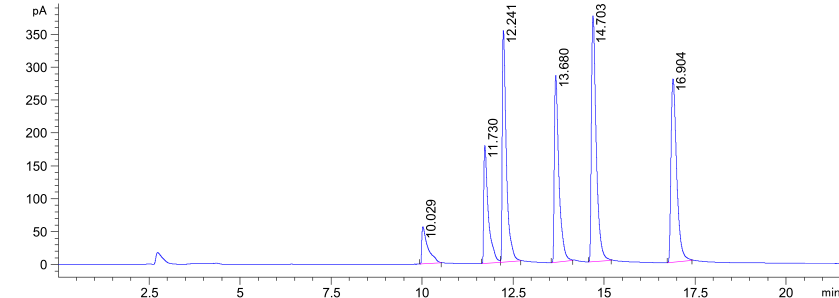


**Table S1** The ingredient compositions of the experimental diets (g/kg Diet)

|  | HF(g/kg) | HFC(g/kg) | HFBM(g/kg) |
| --- | --- | --- | --- |
| Corn starch | 449.5 | 449.5 | 399.5 |
| Lard | 100 | 100 | 100 |
| Inulin | 0 | 0 | 50 |
| Soybean oil | 40 | 40 | 40 |
| a AIN-76 mineral mixture (%mixture) | 35 | 35 | 35 |
|  |  |  |  |
| Casein | 200 | 200 | 200 |
| b AIN-76 vitamin mixture (%mixture) | 10 | 10 | 10 |
| Sucrose | 100 | 100 | 100 |
| L-cystine | 3 | 3 | 3 |
| Cellulose | 50 | 50 | 50 |
| Choline Chloride | 2.5 | 2.5 | 2.5 |
| Cholesterol | 10 | 10 | 10 |
| Capsaicin |  | 0.01 | 0.01 |

**Table S2** Calibration curves of different short-chain fatty acids (SCFAs) in standard solution

| SCFAs | Linear equation | Correlation coefficient (R^2^) |
| --- | --- | --- |
| Acetic acid | y=62.583x-795.68 | 0.9914 |
| Propionic acid | y=121.56x-1144.4 | 0.9963 |
| i-butyric acid | y=171.47x-942.01 | 0.9988 |
| n-butyric acid | y=171.6x-1250.1 | 0.998 |
| i-valeric acid | y=193.82x-893.63 | 0.9996 |
| n-valeric acid | y=204.8x-843.75 | 0.9997 |
